# Use of Artificial Neural Networks for Recycled Pellets Identification: Polypropylene-Based Composites

**DOI:** 10.3390/polym17172349

**Published:** 2025-08-29

**Authors:** Maya T. Gómez-Bacab, Aldo L. Quezada-Campos, Carlos D. Patiño-Arévalo, Zenen Zepeda-Rodríguez, Luis A. Romero-Cano, Marco A. Zárate-Navarro

**Affiliations:** 1 Laboratorio de Ingeniería Química, Departamento de Biotecnológicas y Ambientales, Universidad Autónoma de Guadalajara, Av. Patria 1201, Zapopan CP. 45129, Jalisco, Mexico; maya.gomez@edu.uag.mx (M.T.G.-B.); aldo.quezada@edu.uag.mx (A.L.Q.-C.); 2 Grupo de Investigación en Materiales y Fenómenos de Superficie, Departamento de Biotecnológicas y Ambientales, Universidad Autónoma de Guadalajara, Av. Patria 1201, Zapopan CP. 45129, Jalisco, Mexico; carlos.patino@edu.uag.mx (C.D.P.-A.); luis.cano@edu.uag.mx (L.A.R.-C.); 3 Institute of Polymer Science and Technology (CSIC), Elastomers Group, C/Juan de la Cierva 3, 2800 Madrid, Spain; zenen@ictp.csic.es

**Keywords:** polymer recycling, artificial neural network, sustainability, composite classification

## Abstract

Polymer recycling is challenging due to practical classification difficulties. Even when the polymer matrix is identified, the presence of various polymeric composites complicates their accurate classification. In this study, Fourier-transform infrared spectroscopy (ATR-FTIR) was used in combination with artificial neural networks (ANNs) to quantitatively predict the mineral filler content in polypropylene (PP) composites. Calibration curves were developed to correlate ATR-FTIR spectral features (600–1700 cm^−1^) with the concentration (wt.%) of three mineral fillers: talc (PP-Talc), calcium carbonate (PP-CaCO_3_), and glass fiber (PP-GF). ANN models developed in MATLAB 2024a achieved prediction errors below 7.5% and regression coefficients (R^2^) above 0.98 for all filler types. The method was successfully applied to analyze a commercial recycled pellet, and its predictions were validated by X-ray fluorescence (XRF) and energy-dispersive X-ray spectroscopy (EDX). This approach provides a simple, rapid, and non-destructive tool for non-expert users to identify both the type and amount of mineral filler in recycled polymer materials, thereby reducing misclassification in their commercialization or quality control in industrial formulations.

## 1. Introduction

Plastics have become an indispensable part of modern life, serving as essential materials in industry, packaging, construction, and agriculture. However, the rapid increase in plastic production is leading to an equally alarming rise in plastic waste, with a projection of 460 million tons by 2030 [1]. This unprecedented accumulation poses threats to the environment, including air, water, and ecosystem pollution, and contributes to climate change through carbon emissions associated with polymer-derived fossil fuels [2]. Mismanagement of plastic waste globally has resulted in a multitude of environmental issues, which could be tackled by boosting plastic recycling rates [3]. Plastic recycling for composites production does offer environmental advantages for certain applications, while, in others, recycling cannot be performed due to intense degradation [4,5,6].

Recycling is then a crucial strategy for mitigating the environmental impact of plastic waste, and the transition from neat plastic to recycled is a crucial step [7], although this is a multifactorial process, involving social and technological aspects [8,9]. Despite challenges such as the degradation of material quality in mechanical recycling or the high energy requirements of chemical recycling, the promotion of added value solutions presents a more sustainable path forward, aligning with the principles of a circular economy [10]. Materials recovery facilities require new automated technologies if growing recycling demands are to be met [11]. In general, the final properties of polymeric blends will be determined by the concentration of the mixture and the nature of their components. Particularly, the extensive use of polypropylene (PP) in various industries necessitates the development of efficient and reliable methods for predicting the mechanical properties of PP and its composites [12], glass transition temperature [13], and milling temperature of composites [14]. A more conscious control has motivated some studies to quantify and/or classify these materials according to the concentration of their components. The fully recycled PP–glass fiber composites are still able to guarantee high final performances and can be successfully used for designing new structural components [5], with a mechanical performance comparable to that of the neat counterpart, or even their separation using solvents [15,16,17], which results in an almost neat PP, since low quantities of inorganic fillers have almost no impact on toughness [18]. Another common mineral filler is calcium carbonate, with similar findings for this composite [19].

Despite their use, traditional methods such as chemical and mechanical recycling often face challenges related to their efficiency, purity, and processing speed [20]. Economic viability should also be a focus, with a review of the cost–benefit dynamics of using recycled thermoplastics, considering factors such as production costs, market demand, and barriers to adoption. Integrating thermoplastic waste with bio-based or hybrid materials, including natural fibers, presents another potential research direction, enhancing the sustainability and performance of composites [21,22]. Thus, a common approach to recycled polymers is the production of material composites, either with inorganic fillers or natural fibers [22,23,24] or blending with other polymers [25].

On the other hand, spectrometry techniques, such as Fourier-transform infrared spectrometry (FTIR) and near-infrared spectroscopy, have proven capable of overcoming these limitations. These methods allow for the non-destructive, accurate, and rapid analysis of polymeric materials, facilitating the qualitative and quantitative characterization required for their effective sorting and recycling [26,27]. IR spectroscopy is the most widely applied spectroscopic method for chemometric analysis for plastic waste sorting [3]. Current optical screening devices use visible and near-infrared (NIR) wavelengths, frequency ranges that can experience challenges during the characterization of postconsumer plastic waste because of the overly absorbing spectral bands from dyes and other polymer additives [11]. However, conventional methods also face challenges, especially in the management of random and unknown factors, such as inhomogeneous compositions, poor casting or extrusion, and non-compact pellets, among others, which affect the polymer composition and can lead to erroneous data; therefore, the use of a more efficient and reliable method is necessary [28,29]. Advances in databases and machine learning algorithms are making polymer informatics more efficient and accessible; nonetheless, continuous improvement is needed.

In this sense, in [30], a plastic package classification was performed by combining middle-range infrared analysis along with independent component analysis, a method of blind source separation, extracting independent source signals from a set of signals where they are mixed in unknown proportions, by looking for a linear transformation that maximizes the statistical independence of the components. The estimated independent components are often closely related to the spectral profiles of the individual chemical components in the initial mixture and thus chemically interpretable.

Advances in machine learning, particularly convolutional neural networks, have fostered an increase in the potential of spectrometry techniques. Machine learning is a data analysis method that has proven to be effective for polymer classification using NIR and FTIR [1]. The required data pursue authenticity, comprehensiveness, and objectivity. The learning process of a model is to adjust the internal parameters in a function that, given a specific set of input values, calculates the acceptable output values. After the parameter adjustment based on data is completed, the model can calculate and predict the output values of new samples [31]. The typical approach in machine learning involves splitting the dataset into a training set used to train the model and a test set used to evaluate its performance.

By automating data extraction and analysis, these models enable more accurate and efficient identification of polymer types and compositions. Several studies have shown their effectiveness of combining spectrometry and machine learning to classify polymer mixtures and analyze their composition with high precision [32]. Artificial neural networks (ANNs) have demonstrated superior performance in the prediction and optimization of machining processes when compared to traditional methods. Their ability to model complex, nonlinear relationships between process parameters enables them to deliver more accurate and reliable predictions, making them highly effective in optimizing machining outcomes. Its capability to predict the behavior of complex nonlinear structural systems while considering a wide range of parameters offers a distinctive opportunity with several analytical applications, such as calcium carbonate content in cement paste [33] or even alcoholic beverages [34]. ANN models have been frequently employed to predict mechanical properties using extensive datasets that cover a large scale of input variables. This is due to the nature of ANN models’ flexibility, nonlinearity, scalability, and ability to learn from extensive datasets [35]. Regarding polymer classification, the ATR-FTIR spectroscopy chemometric data have shown to be useful even when classifying recycled black plastics [36].

This contribution focuses on the use of ANNs to develop a quantitative methodology for analyzing polypropylene composites through FTIR spectrometry, improving the efficiency of the recycling process or for industrial formulations from recycled polypropylene, promoting both sustainability and ensuring quality control.

## 2. Materials and Methods

The methodology involves preparing a series of polymer composites with systematically varied mineral filler concentrations, followed by ATR-FTIR spectral acquisition. Key spectral features (e.g., peak height, width, and center) were extracted and utilized as input variables for the artificial neural networks, with the corresponding known filler concentrations serving as the output. The method was evaluated using both commercial samples and an independently sourced reference spectrum. Validation was performed through complementary characterization techniques. ANN performance was quantitatively assessed using standard metrics. The detailed experimental procedures are provided in subsequent sections.

### 2.1. Sample Preparation

To obtain spectral data for the calibration curve, various powder–powder mixtures were prepared, combining polymer and mineral filler at different concentrations. The polymer matrix was composed of neat polypropylene from Formosa Plastics corporation (Livingston, NJ, USA); for its preparation, the pellets were frozen at −70 °C, followed by pulverizing the material using a grinder and then sieving it to obtain a homogeneous particle size distribution. The mixture was then melted to integrate the solute into the polymeric matrix. The mineral fillers analyzed consisted of calcium carbonate from Sigma-Aldrich (Toluca, Mexico), talc from TEMISA S.A. de C.V (Tlaquepaque, Mexico), and glass fiber from Corning Glass Works (Corning, NY, USA). The concentrations at which the mixtures were made are as follows: 5, 10, 15, 20, 30, and 40% wt.

Additionally, three real samples of composite materials provided by RECO Recycling group (Zapotlanejo, Mexico) or recycled pellets (PP-Talc 30% and PP-GF 20% samples) and one provided by Prof. Techawinyutham Lab (PP-CaCO_3_ 10% wt. sample) were analyzed in order to validate the proposed ANNs.

### 2.2. Analytical Equipment and Measurements

After sample preparation, a Nicolet iS5 Family Spectrometer (Thermo Fisher Scientific, Waltham, MA, USA) was used to perform the measurements. Four measurements were taken for each composition. This is especially important for commercial samples, since some mineral fillers may liberate gases, causing bubbles inside the pellet (see Figure S1). The FTIR spectrum was divided into two regions: one corresponding to the identification of the polymer, in an approximate range of wave numbers between 4000 and 1500 cm^−1^, while, in the approximate range of wave numbers between 1500 and 400 cm^−1^, there are signals corresponding to the mineral charge. The analytical validation of the instrument was carried out by evaluating the consistency and reproducibility of the results against one standard (urea; Figure S2) for which a similar percentage of 100% was obtained with respect to the Thermo Fisher library.

### 2.3. Physicochemical Characterization of Commercial Samples

The actual samples provided by the manufacturer were analyzed by scanning electron microscopy using a Desktop SEM, SNE-Alpha Scanning Electron Microscope (Suwon, Republic of Korea) to evaluate their morphology and the dispersion of the inorganic filler. Subsequently, the elemental composition was evaluated by energy-dispersive X-ray spectroscopy (EDX) in selected regions of the sample surface. The analysis was performed using an EDX detector Hitachi SU8000 (Tokyo, Japan), and Bruker nano xflash detector 5030 (Berlin, Germany), 15 kv, 8 kvt. To isolate the inorganic fraction, a calcination step was performed at 700 °C for 2 h. The remaining residue was subjected to X-ray fluorescence analysis using an Epsilon 4 spectrometer from Malvern Panalytical (Malvern, United Kingdom) to quantify the elemental composition of the mineral filler. Finally, X-ray diffraction (XRD) was used to identify the crystalline phases present in the residue using a Bruker D2 PHASER (Karlsruhe, Germany) with Cu Kα radiation (λ = 1.5406 Å), operating at 30 kVA and 10 mA. The diffractograms were obtained in a 2θ range from 0° to 80° with a 0.01° step.

### 2.4. Artificial Neural Network Approach

**Figure 1:**
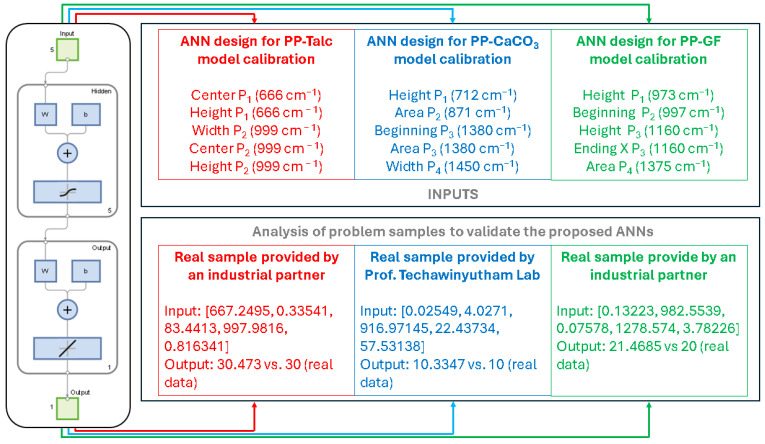
Architecture of the proposed artificial neural networks (ANNs) with their respective inputs, or chemometric fingerprints, for predicting the mineral filler (talc, CaCO_3_, and glass fiber) in polypropylene-based composites, as well as an example of its use for prediction in real samples.

A MATLAB 2024a (The MathWorks Inc., Natick, MA, USA) script that employs an artificial neural network (ANN), specifically a feedforward neural network (FFNN) trained with backpropagation and Bayesian regularization, has been used to estimate the concentration of an unknown sample based on a nonlinear calibration curve (inputs), leveraging a dataset of the observations with the selected input features, that is, the spectral data of the composites, and using the mass fraction as the output. The methodology begins by normalizing both input and output data to ensure consistent scaling, using z-score normalization to center the data around zero with unit variance, which is critical for ANN performance. A Bayesian regularization training function (trainbr) is selected to mitigate overfitting, a common risk with small datasets, as it automatically penalizes overly complex models. In this sense, we conceive a relatively small dataset as a number of observations near the minimum required by the MATLAB tools, that is, 11 observations, which has been dealt with in material and biomedical sciences, since data acquisition is expensive and time-consuming [37,38]. Of course, this approach can benefit from a bigger dataset, in composition, filler type, or other matrices, since ANN can handle these multivariate fitting problems with exceptional performance. Cross-validation (5-fold) is integrated to robustly evaluate model performance by partitioning the data into training and validation subsets, ensuring the model generalizes well despite limited samples. The ANN architecture comprises an input layer (5 neurons), one hidden layer (5 neurons, tanh activation), and a linear output neuron, balancing simplicity and predictive power in regression tasks (Figure 1). After training, the model processes the unknown sample by first normalizing its input features using the calibration dataset’s statistics, then predicting the concentration in normalized space, and, finally, de-normalizing or mapping the output to the original scale. This approach ensures accuracy, minimizes overfitting, and accounts for nonlinear relationships, making it particularly suitable for small, high-dimensional datasets where traditional regression methods may underperform.

To complement the ANN description, Figure 1 summarizes the model architecture and highlights the selected spectral fingerprints (wavenumbers, cm^−1^) used as inputs for the three calibration models (PP-Talc, PP-CaCO_3_, and PP-GF). The lower panels illustrate representative predictions of real samples compared to experimental values, demonstrating the ANNs’ capability to accurately estimate the concentration even with limited datasets.

## 3. Results and Discussion

### 3.1. Spectral Fingerprint of Neat Polypropylene and Its Implications for Filler Quantification

The ATR-FTIR spectrum of neat polypropylene (PP), shown in Figure S2, displays the characteristic absorption bands associated with the vibrational modes of the polymer matrix. Notably, strong absorption peaks appear at ~2950–2840 cm^−1^, which correspond to the asymmetric and symmetric stretching vibrations of the CH_3_ and CH_2_ groups, respectively. These bands are typical of aliphatic hydrocarbons and are commonly observed in saturated polyolefins such as PP. Additionally, the bands observed at ~1455 cm^−1^ and ~1375 cm^−1^ can be attributed to CH_2_ and CH_3_ bending vibrations, respectively [39].

In the region below 1200 cm^−1^, the spectrum of neat PP shows relatively low-intensity features, consistent with the absence of polar functional groups or mineral content in the pure polymer matrix. This region, particularly between 1200 and 600 cm^−1^, is highly sensitive to inorganic fillers such as talc, calcium carbonate, and glass fibers, which exhibit characteristic Si–O stretching (~1000 cm^−1^), Mg–O bending (~670 cm^−1^), and carbonate (CO_3_^2−^) out-of-plane deformations (~870 and 710 cm^−1^), respectively. These vibrational modes arise from the intrinsic molecular structures of the filler particles, and their presence introduces distinct, sharp absorption bands in the otherwise featureless spectrum of the polymer. In addition to their spectral signatures, fillers can alter the microstructure and density of the composite material, leading to subtle shifts in peak positions or intensities due to matrix–filler interactions.

The near-zero absorbance values in the fingerprint region (<1200 cm^−1^) for neat PP are of critical importance, as they provide a clean spectral baseline [39]. This contrast enables clear differentiation once inorganic additives are introduced and allows these low-absorbance zones to serve as analytical windows for constructing calibration curves. The well-defined filler-related bands in this region serve as reliable spectral descriptors, which can be extracted and used as inputs for artificial neural network (ANN) models aimed at quantifying filler content with high sensitivity.

### 3.2. Construction of ANNs for the Prediction of Talc, CaCO_3_, and Fiberglass Content in Polypropylene-Based Composites

**Figure 2:**
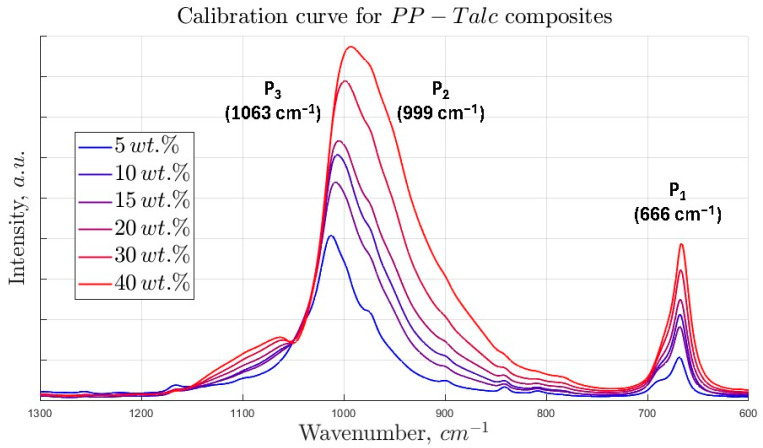
ATR-FTIR spectra of the prepared PP-Talc composite samples at different concentrations of the mineral filler.

The ATR-FTIR spectra of the polypropylene–talc composites are presented in Figure 2. Three characteristic absorption bands, labeled as P_1_ (666 cm^−1^), P_2_ (999 cm^−1^), and P_3_ (1063 cm^−1^), are clearly identified and correspond to vibrational modes associated with the talc filler. Specifically, P_1_ is attributed to the Mg–O stretching vibration in the octahedral sheets of the talc structure, P_2_ is associated with Si–O–Si bending vibrations, and P_3_ corresponds to Si–O stretching modes within the silicate tetrahedra [40].

**Figure 3:**
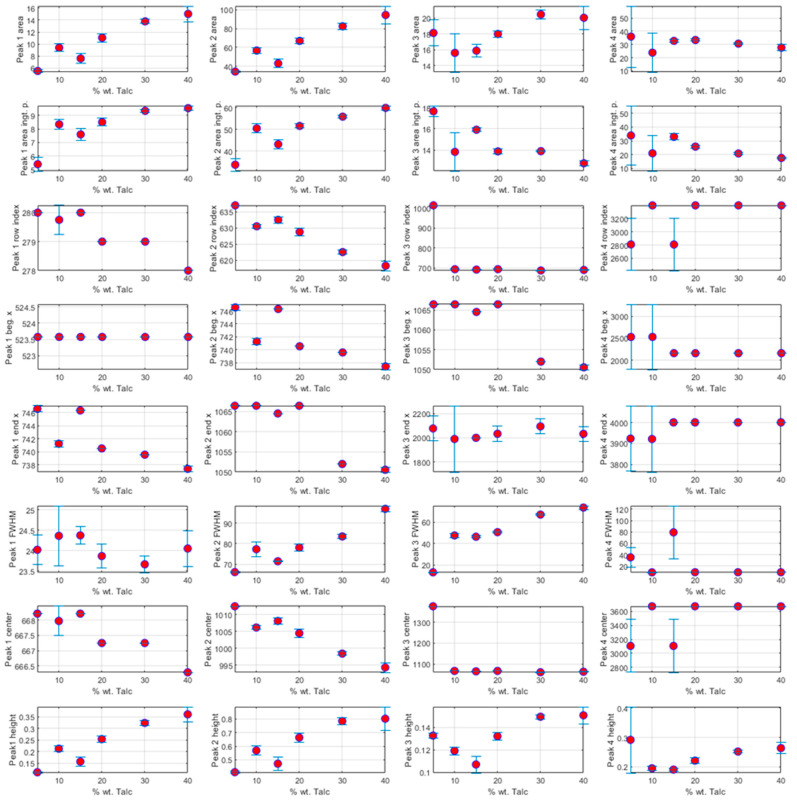
Correlation analysis between selected ATR-FTIR spectral features and mineral filler content (Talc), highlighting the correlations used for ANN input selection. Each point is the average value of the chemometric feature at a given concentration, displaying the error bars to indicate variability.

The characterization of each peak (Figure 3) was performed by extracting the following spectral parameters: (i) *Area* (obtained by integrating the peak signal from the baseline); (ii) *AreaIntgP* (percent area, representing the relative contribution of each peak to the total area under the curve); (iii) *Row Index* (the index of the data point where the peak center occurs); (iv) *Beginning X* and (v) *Ending X* (the wavenumber positions corresponding to the leftmost and rightmost points of each peak, respectively); (vi) *FWHM* (full width at half-maximum, indicating the peak width at half of its maximum intensity); (vii) *Center* (the wavenumber at the peak maximum); and (viii) *Height* (the intensity at the peak maximum relative to the baseline). The baseline was set at the minimum intensity value across the spectral region of interest and remained constant across all samples.

Based on this analysis, it was concluded that a robust correlation between talc concentration and ATR-FTIR spectral features can be established by jointly evaluating the *Center* and *Height* of Peak 1 (around 666 cm^−1^), along with the *width*, *center*, and *height* of Peak 2 (near 999 cm^−1^). These specific spectral characteristics were selected due to their strong and consistent dependence on the talc content across the tested samples. While parameters such as *Row Index* and *Area* also exhibited a similar trend, they were excluded from the optimal feature set to avoid redundancy with the *Center* and *Height* variables and to reduce the risk of overfitting in the ANN model. Therefore, only the most informative and non-collinear spectral parameters were retained as essential predictors for estimating the talc content in the polypropylene composites. On the other hand, the correlation matrices of the chemometric features are found in Table S2, known as Pearson matrices [41]. According to our experience, we have defined a correlation threshold of $\left|0.98\right|$ in order to preserve the relevant features. In this sense, reducing the number of variables to optimize is rather a subjective task [42].

**Figure 4:**
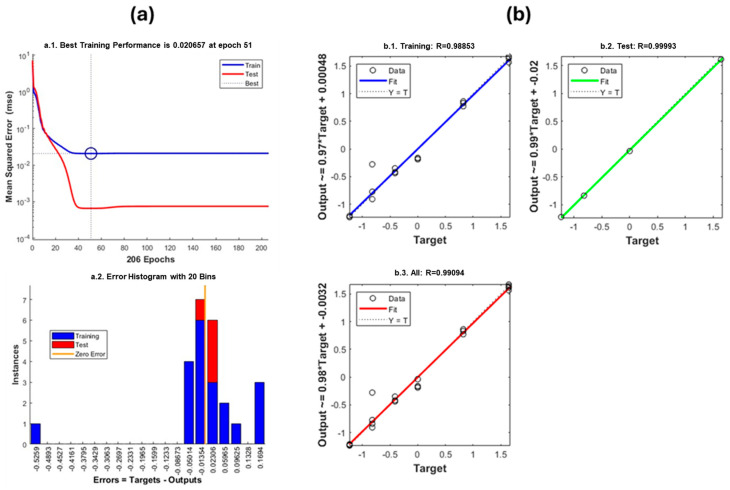
Performance of the ANN for talc content prediction. (**a**) Model performance metrics: mean squared error (MSE) progression during training and testing (normalized units) and the distribution of prediction errors. (**b**) Regression plot comparing predicted versus actual values, demonstrating model accuracy with high correlation (R > 0.98) and minimal prediction error for all data subsets.

The previously selected ATR-FTIR spectral features were used as input variables in the architecture of the proposed artificial neural network (ANN). The training process was conducted using a supervised learning approach with backpropagation, employing a dataset constructed from composites of known filler concentrations and their corresponding spectral features. The network was trained to predict the talc content based on these inputs, and its performance was evaluated using standard regression and error metrics (Figure 4).

The ANN achieved the best training performance with a mean squared error (MSE) of 0.020657 at epoch 51 (normalized scale). Both the training and test errors decreased rapidly during the initial epochs and stabilized without divergence, indicating no evidence of overfitting. The error histogram further confirmed that most prediction errors were centered near zero with a symmetrical distribution, supporting the accuracy and stability of the model.

**Figure 5:**
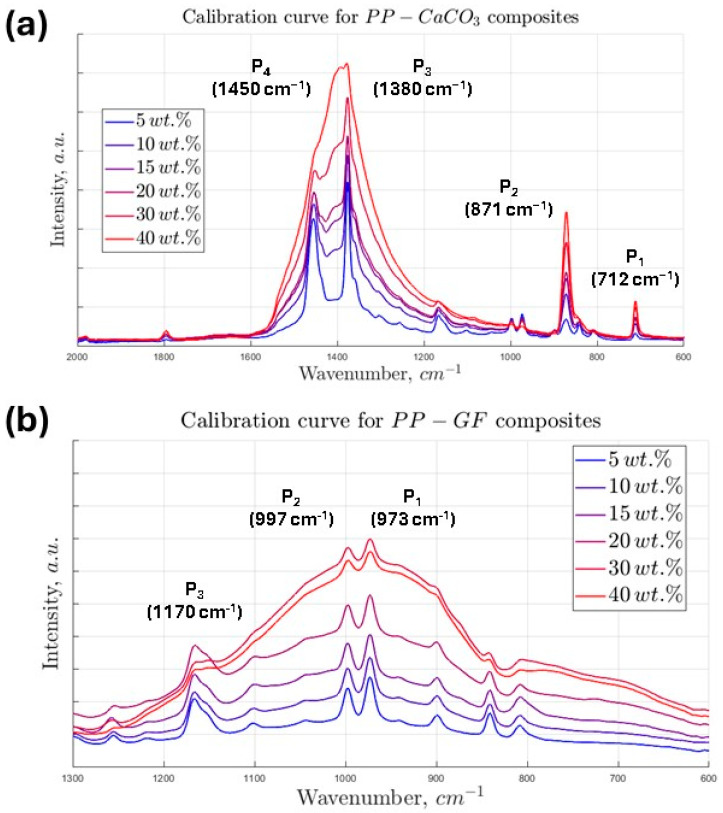
ATR-FTIR spectra of (**a**) PP-CaCO_3_ and (**b**) PP-GF composite samples at different mineral fill concentrations.

A similar analytical procedure was applied to the characterization of the polypropylene composites containing calcium carbonate (PP-CaCO_3_) and glass fiber (PP-GF), with the aim of validating the ANN-based predictive framework across different filler types. In the case of the PP-CaCO_3_ composite, the ATR-FTIR spectra (Figure 5a) revealed four prominent absorption bands located at P_1_ = 712 cm^−1^, P_2_ = 871 cm^−1^, P_3_ = 1380 cm^−1^, and P_4_ = 1450 cm^−1^, which are consistent with vibrational modes of carbonate species [43]. Specifically, the bands at 712 and 871 cm^−1^ are attributed to out-of-plane and in-plane bending vibrations of the CO_3_^2−^ anion, respectively, while the bands at 1380 and 1450 cm^−1^ correspond to symmetric and asymmetric stretching modes of the carbonate group. As with the talc-based composite, a mathematical analysis of these peaks was performed to extract spectral descriptors (Figure S3). Through correlation analysis, the most informative features were identified as the *Height* of P_1_, *Area* of P_2_, *Beginning* and *Area* of P_3_, and *Width* of P_4_. These features were used as input variables for the corresponding ANN model trained to predict the calcium carbonate content.

In the case of the PP-GF (glass fiber) composite, the ATR-FTIR spectrum exhibited four distinct absorption peaks centered at P_1_ = 973 cm^−1^, P_2_ = 997 cm^−1^, P_3_ = 1160 cm^−1^, and P_4_ = 1375 cm^−1^, which are associated with Si–O stretching vibrations in the silicate network of glass fibers [44]. Following the same peak analysis and parameter extraction procedure, the features selected for model input included the *Height* P_1_, *Beginning* P_2_, *Height* P_3_ and *Ending* P_3_, and *Area* P_4_ due to their clear linear dependence on the filler concentration. These features were fed into the ANN model specifically trained on glass fiber composites.

For both PP-CaCO_3_ (Figure S4) and PP-GF (Figure S6) composites, the performance of the ANNs using the selected input features was comparable to that previously discussed for the talc-based system. The training curves demonstrated stable convergence, the regression plots showed high correlation coefficients (R > 0.99), and the error histograms exhibited a narrow and symmetric distribution centered near zero. These consistent results reinforce the reliability of the FTIR-based ANN models across different filler types and concentrations. Calibration curves were derived from additive-free polymer–filler systems. Additives, such as antioxidants and stabilizers, among others, are typically incorporated at low concentrations (<2 wt.%) and have been reported in non-interfering spectral bands [45,46,47]. Even in cases of potential overlap, the polymeric matrix is maintained constant, and the additions are only of mineral fillers. The proposed method is robust, demonstrated by the successful identification of the commercial samples, which do contain additives. Organic fillers such as cellulose or fibers are not considered in this study. A potential extension to this characterization is to train the ANN to determine the degradation state of the polymer, since it is known that extrusion cycles impact the mechanical properties [48].

The predictive performance of the ANN was benchmarked against other established machine learning methodologies, including Ensemble Methods, Support Vector Regression (SVR), and Gaussian Process Regression (GPR), on the PP-CaCO_3_ dataset. Evaluation via five-fold cross-validation demonstrated the ANN’s superior performance, yielding a mean squared error (MSE, original scale) of 17.67 and a coefficient of determination of R^2^ = 0.986. In contrast, Ensemble Methods—specifically, Random Forest (fitrensemble, “Bag”) and Gradient Boosting (fitrensemble, “LSBoost”)—exhibited higher error rates, with MSEs (original scale) of 27.95 and 36.25, and corresponding R^2^ values of 0.827 ± 0.073 and 0.705 ± 0.232, respectively. Gaussian Process Regression (fitrgp) demonstrated competitive performance akin to the ANN, while Support Vector Regression (fitrsvm) resulted in a substantially higher estimation error exceeding 40%.

Although the ANN model achieved the most favorable metrics in this specific application, it is imperative to avoid the generalized conclusion that a single algorithm is universally superior. Model performance is inherently influenced by stochastic elements during training and is highly contingent upon dataset characteristics, such as size, dimensionality, and noise structure, as well as the specific modeling objectives [49,50,51]; consequently, the optimal algorithm is often context-dependent. Besides its predictive accuracy, the ANN framework presents a compelling advantage due to its straightforward implementation in MATLAB, making it a practical and efficient choice for our application.

Collectively, these results confirm that the proposed FTIR–ANN framework is broadly applicable for the quantification of a variety of inorganic fillers in polypropylene matrices. The strategy demonstrates strong predictive power, minimal error rates, and excellent generalization capabilities across different types of fillers, including talc, calcium carbonate, and glass fiber, without requiring extensive retraining. This supports its use as a practical and non-destructive analytical tool for rapid material characterization in industrial polymer processing and quality control contexts, and although the processing conditions require mechanical and rheological information that is out of the scope of this contribution, they are fundamentally dependent on the composition; thus, our method is complementary to ensure the final user the properties they expect [52].

### 3.3. Use of the Proposed ANNs for the Study of Commercial Samples

**Figure 6:**
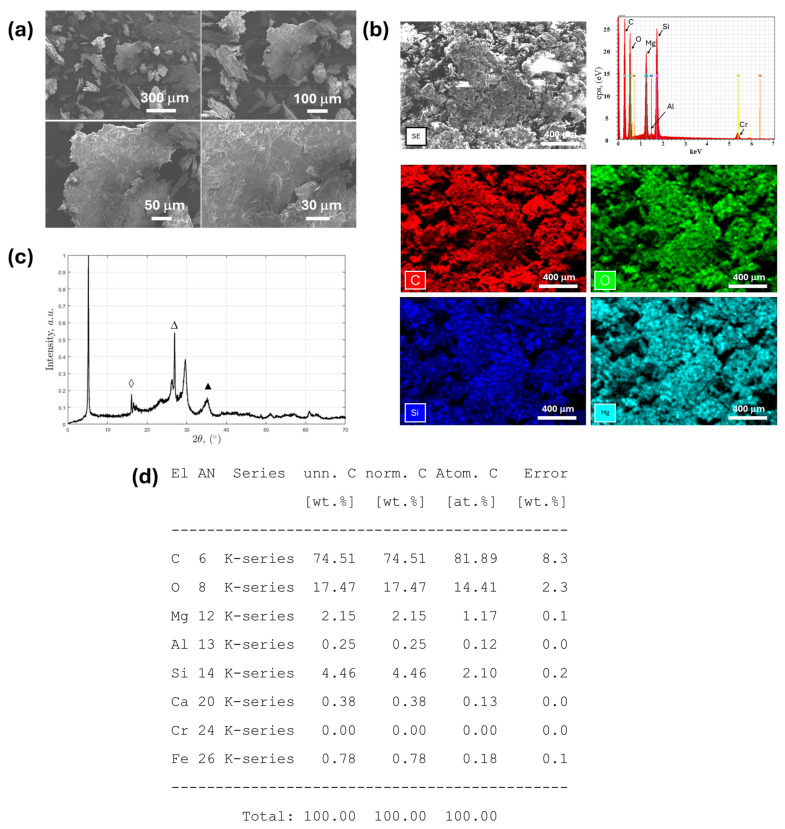
Physicochemical characterization of the PP-Talc30% sample provided by a local industry: (**a**) SEM micrographs, (**b**) SEM-EDX mapping, (**c**) diffractogram of the calcined sample; crystallographic planes of talc: ◊ (002), ∆ (004), ▲ (006), and (**d**) semiquantitative EDX analysis.

The polymeric composite sample provided by the manufacturer, declared to contain 30 wt.% talc as a filler and polypropylene as the polymeric matrix, was first examined by scanning electron microscopy (SEM) to assess its morphological characteristics. The SEM micrographs (Figure 6a) revealed a homogeneous microstructure, with dispersed lamellar or plate-like particles embedded within a continuous polymeric matrix. These lamellar features are consistent with the typical morphology of talc, a phyllosilicate mineral known for its platy crystallite habit. The talc particles appeared well distributed within the polypropylene matrix, with some degree of agglomeration observed in localized areas [53].

To verify the chemical composition and confirm the presence of talc, SEM-EDX analysis was performed on selected regions of the composite. The EDX spectrum (Figure 6a,d) of the polymeric material showed a dominant carbon signal (74.51 wt.%), as expected for a polypropylene-based matrix, accompanied by oxygen (17.47 wt.%), silicon (4.46 wt.%), and magnesium (2.15 wt.%) signals. These latter elements are characteristic of talc (Mg_3_Si_4_O_10_(OH)_2_), confirming its presence in the analyzed region [54]. Minor elements such as aluminum (0.25 wt.%), calcium (0.38 wt.%), and iron (0.78 wt.%) were also detected in trace amounts, potentially originating from mineral impurities or additives. Although the EDX analysis revealed slightly lower-than-expected concentrations of Mg and Si, this apparent discrepancy is attributed to the localized nature of the measurement and the matrix attenuation effect inherent to polymer-rich areas.

To eliminate the influence of the polymeric phase and gain a clearer understanding of the inorganic content, the composite was subjected to thermal treatment to remove the polypropylene component by calcination. The resulting residue was then analyzed by X-ray fluorescence, XRF (Table S1), X-ray diffraction, and XRD (Figure 6c). The XRF analysis of the calcinated material revealed a MgO content of 18.381 wt.%, which corresponds to approximately 57.6 wt.% talc in the inorganic fraction when calculated based on the stoichiometric MgO content in pure talc. This result is in good agreement with the original 30 wt.% talc content declared by the manufacturer, considering that the organic matrix has been removed.

Finally, the XRD pattern of the calcinated residue displayed a series of well-defined diffraction peaks that match the characteristic reflections of crystalline talc. Notably, intense peaks at approximately 9.3°, 18.8°, and 28.8° (2θ) correspond to the (002), (004), and (006) planes of talc, respectively, confirming their presence as the main crystalline phase [55]. Additional minor peaks suggested the presence of quartz or other siliceous impurities, in line with the SiO_2_ excess observed in the XRF analysis.

Overall, the combination of SEM imaging, SEM-EDX microanalysis, XRF quantification, and XRD phase identification provided strong evidence that the polypropylene-based composite does contain talc as the major inorganic filler, and the measured content aligns closely with the 30 wt.% value specified by the manufacturer.

Following the morphological, elemental, and structural characterization of the composite, ATR-FTIR spectroscopy was employed to obtain the vibrational fingerprint of the sample. The spectrum was analyzed to extract relevant peak parameters, which were used as input features for a previously trained ANN model developed for talc quantification in polypropylene-based composites. Specifically, mathematical treatment of the ATR-FTIR data allowed for the extraction of peak center, height, and width from two characteristic absorption regions: ~666 cm^−1^ and ~996 cm^−1^. The numerical values used as inputs are *Center* P_1_ = 667.25, *Height* P_1_ = 0.33541, *Width* P_2_ = 83.4413, *Center* P_2_ = 997.98, and *Height* P_2_ = 0.81634. The ANN, which had been trained on a dataset of known concentrations and FTIR-derived features, yielded a predicted talc content of 30.47 wt.%, closely matching the reference value of 30 wt.% provided by the manufacturer. This corresponds to a relative error of only 1.57%, demonstrating excellent predictive performance. Notably, this sample was not included in the training set, indicating that the model generalizes well to unseen real-world data and supporting the robustness and reliability of the ANN-based analytical approach.

A similar analysis was carried out for PP-CaCO_3_ 10% and PP-GF 20% composite samples using their corresponding trained ANN models. The predicted filler contents were 10.3347 wt.% for the PP-CaCO_3_ sample and 21.4685 wt.% for the PP-GF sample, corresponding to relative errors of 3.35% and 7.34%, respectively. In summary, the combination of ATR-FTIR spectral analysis with ANN modeling enabled accurate and non-destructive quantification of inorganic fillers in polypropylene composites. The low prediction errors across multiple filler types—none of which were included in the training datasets—demonstrate the strong generalization ability and practical utility of the proposed methodology for quality control and material verification in polymer-based systems. Although a direct comparison with previously published FTIR–ANN models is not feasible due to differences in input data and modeling strategies, a literature review revealed no other studies combining ANN models with ATR-FTIR spectra for the quantitative prediction of filler content in polypropylene. This underscores the novelty and potential of the proposed method for non-destructive compositional analysis in complex polymer systems.

## 4. Conclusions

It is possible to prepare quantitative calibration curves quickly and reliably, thus streamlining the classification of composite polymer pellets in the recycling industry for quality monitoring purposes. The applications found in MATLAB, such as artificial neural networks, are ideal for this type of task. However, care must be taken with the selection of the inputs and chemometric features to avoid overfitting, as well as with the configuration of the networks, since small datasets are obtained to calibrate the model, along with validation using other instrumental techniques. In this study, early stopping was implemented to prevent overfitting, and the model showed a strong generalization capability, as evidenced by the high regression coefficients (R = 0.99) and a narrow error distribution centered around zero. These results support the robustness of the model, even when trained on a limited dataset. These tools can be exported to Simulink environments, with a more visual appeal, or even compiled as standalone *.exe applications, so that non-expert users can easily use them in the industry and avoid sorting errors or to assure quality control, also increasing the database in the process. These classification errors can be costly and, according to the industrial partner, there are cases of rejected cargos with recycled polymers due to improper characterization of the mineral fillers, affecting supply chains and generating economic losses.

## Data Availability

Data are contained within the article or Supplementary Materials.

## Supplementary Materials

The following supporting information can be downloaded at https://www.mdpi.com/article/10.3390/polym17172349/s1: Details on the chemometric features and ANN fitting, macroscopic structure of the commercial pellets, and chemometric features can be found here. Figure S1. Digital image of the PP-talc sample. Although the components are dispersed in the pellet, inhomogeneities are observed, possibly due to gas formation during extrusion. Figure S2. ATR-FTIR spectra of urea (top) and neat polypropylene (bottom) samples. Figure S3. Correlation analysis between selected ATR-FTIR spectral features and mineral filler content (CaCO_3_), highlighting linear relationships used for ANN input selection. Figure S4. Performance of the ANN for CaCO_3_ prediction: training/test MSE (normalized), error histogram, and regression plots showing high correlation (R > 0.98) and minimal prediction error. Figure S5. Correlation analysis between selected ATR-FTIR spectral features and mineral filler content (glass fiber, GF), highlighting linear relationships used for ANN input selection. Figure S6. Performance of the ANN for glass fiber prediction: training/test MSE (normalized), error histogram, and regression plots showing high correlation (R > 0.95) and minimal prediction error. Table S1. Elemental composition of the polypropylene–talc composite (PP-Talc 30%) determined by X-ray fluorescence (XRF) analysis. Table S2. Pearson correlation matrices of the chemometric features for each polypropylene composite.
