# Supplementary material for: Use of Artificial Neural Networks for Recycled Pellets Identification: Polypropylene-Based Composites
Source: Polymers (Basel). 2025 Aug 29;17(17):2349. doi: 10.3390/polym17172349 (PMC12431023; doi:10.3390/polym17172349)
Supplement: Supplementary file 1 [file polymers-17-02349-s001.zip › polymers-3794910-supplementary.pdf]

polymers

Supplementary Material to Use of Artificial Neural Networks for Recycled Pellets Identification: Polypropylene-Based Composites

Maya T. Gómez-Bacab [1], Aldo L. Quezada-Campos [1], Carlos D. Patiño-Arévalo [2], Zenen Zepeda-Rodríguez [3], Luis A. Romero-Cano [2] and Marco A. Zárate-Navarro [1,*]

[1] Laboratorio de ingeniería química, Departamento de Biotecnológicas y Ambientales, Universidad Autónoma de Guadalajara, Av. Patria 1201, Zapopan CP. 45129, Jalisco, Mexico; maya.gomez@edu.uag.mx (M.T.G.-B.);
aldo.quezada@edu.uag.mx (A.L.Q.-C.)
[2] Grupo de Investigación en Materiales y Fenómenos de Superficie, Departamento de Biotecnológicas y Ambientales, Universidad Autónoma de Guadalajara, Av. Patria 1201, Zapopan CP. 45129,Jalisco, Mexico; carlos.patino@edu.uag.mx (C.D.P.-A.); luis.cano@edu.uag.mx (L.A.R.-C.)
[3] Institute of Polymer Science and Technology (CSIC), Elastomers Group, C/Juan de la Cierva 3, 2800 Madrid, Spain; zenen@ictp.csic.es
* Correspondence: marco.zarate@edu.uag.mx

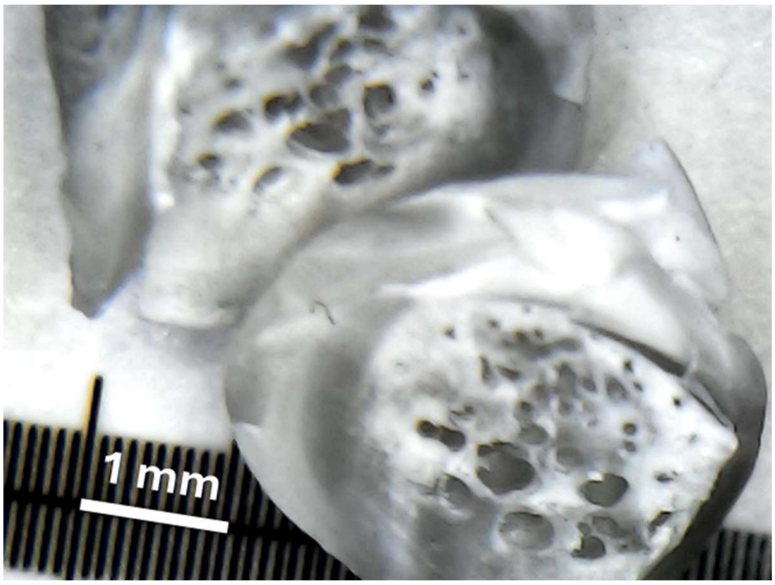

Figure S1. Digital image of the PP-talc sample. Although the components are well dispersed in the pellet, inhomogeneities are observed, possibly due to gas formation during extrusion.

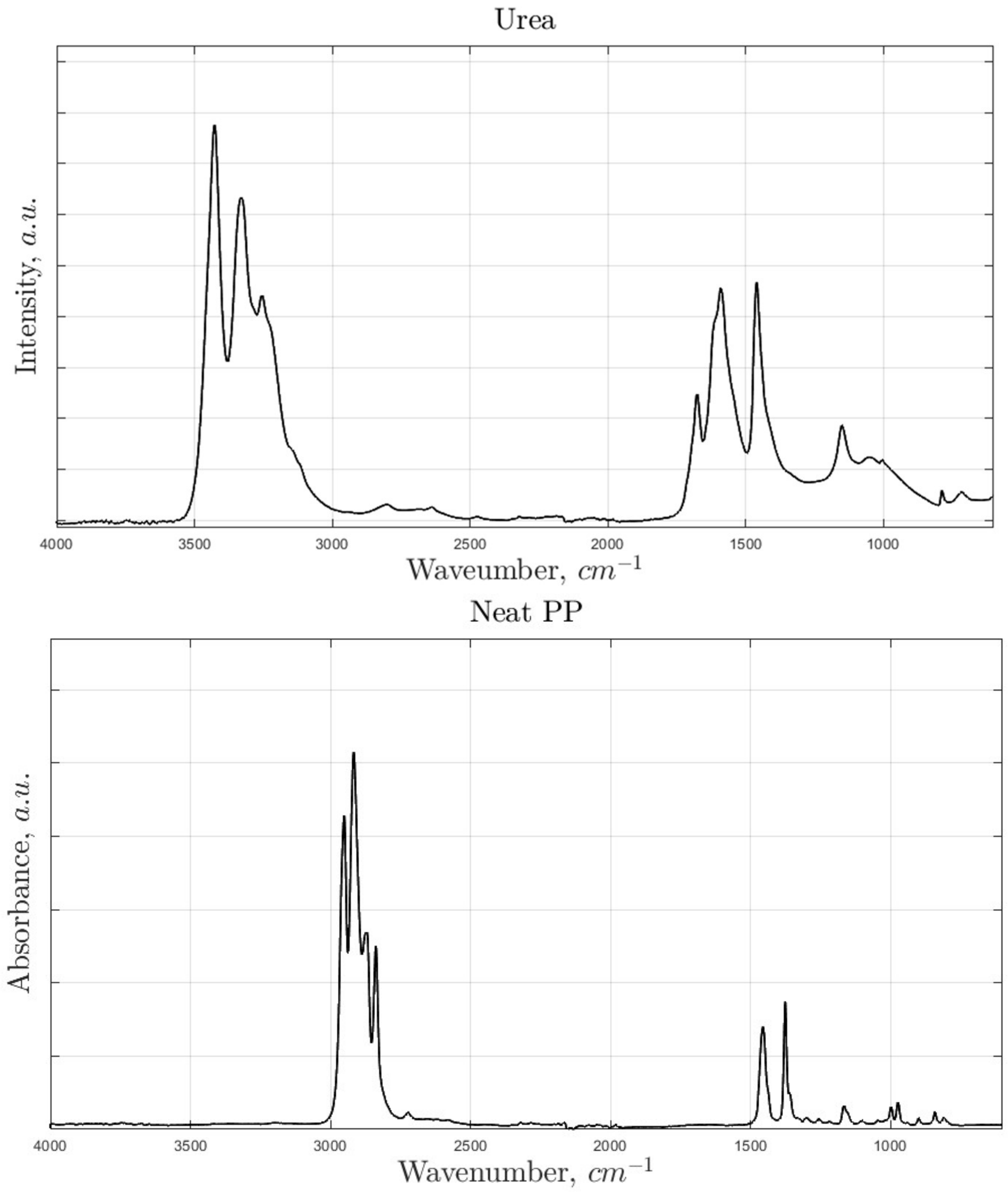

Figure S2. ATR-FTIR spectra of Urea (top) and neat polypropylene (bottom) samples.

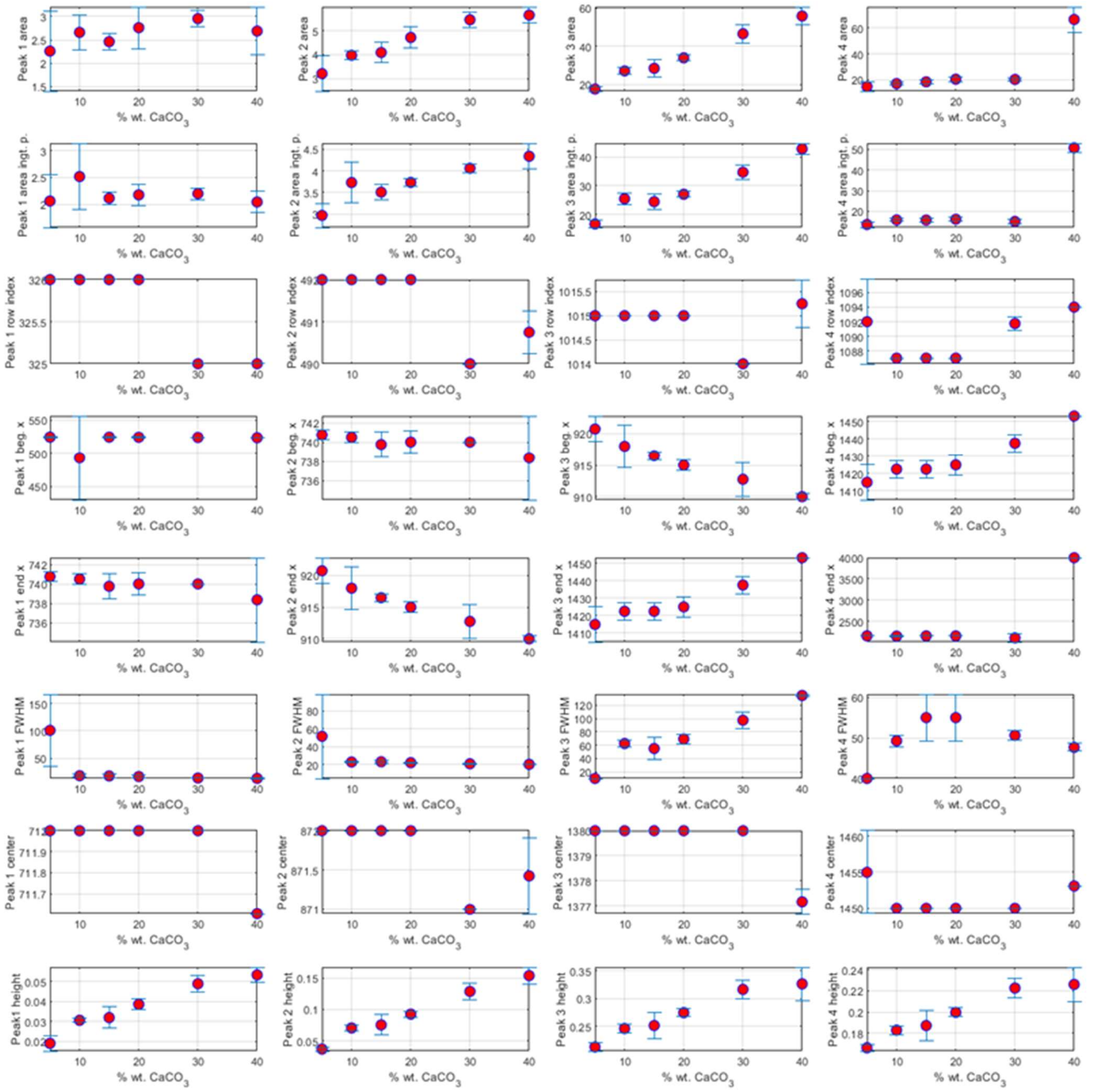

Figure S3. Correlation analysis between selected ATR-FTIR spectral features and mineral filler content ($CaCO_3$), highlighting linear relationships used for ANN input selection.

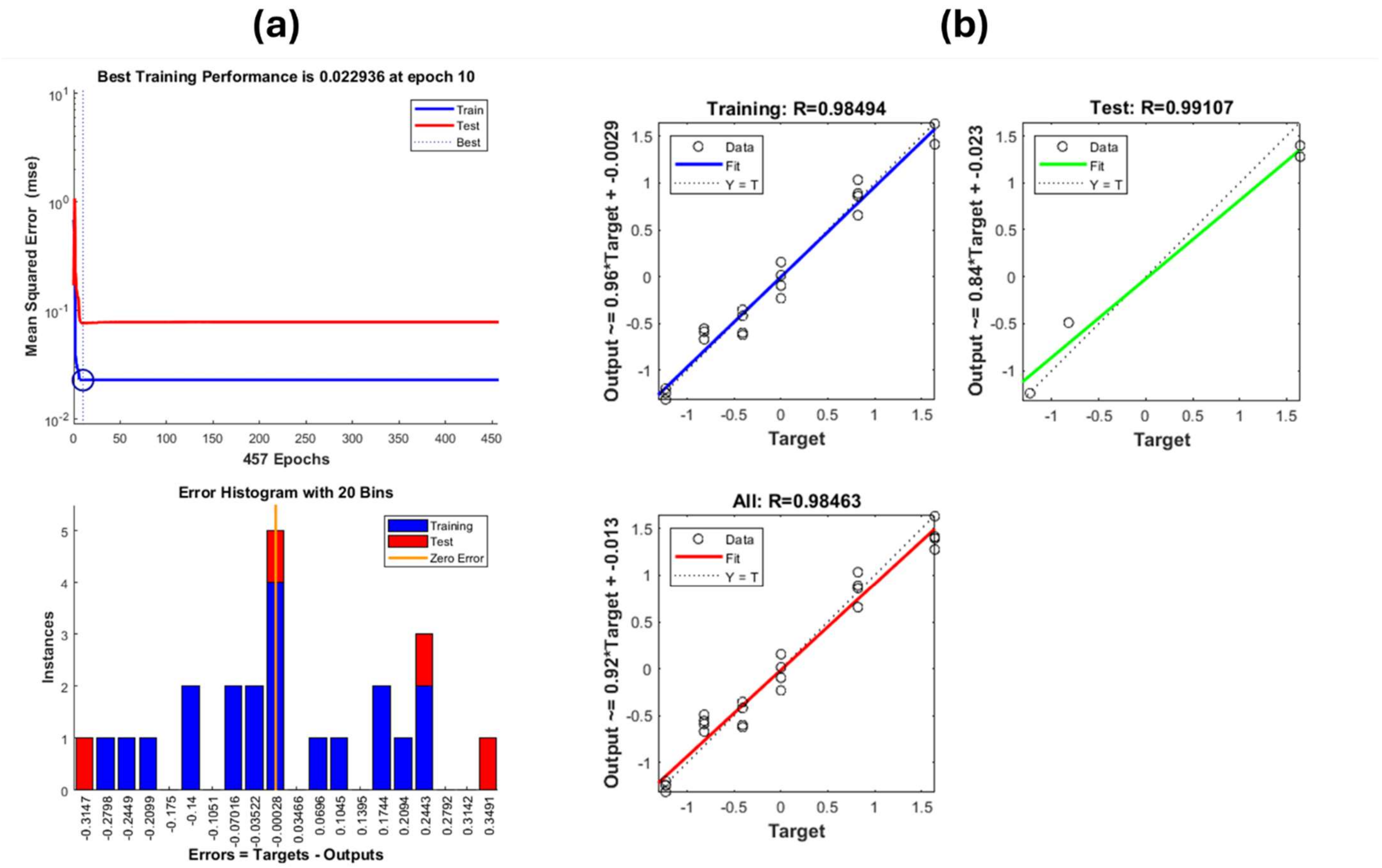

Figure S4. Performance of the ANN for $CaCO_3$ prediction: training/test MSE, error histogram, and regression plots showing high correlation ($R > 0.98$) and minimal prediction error.

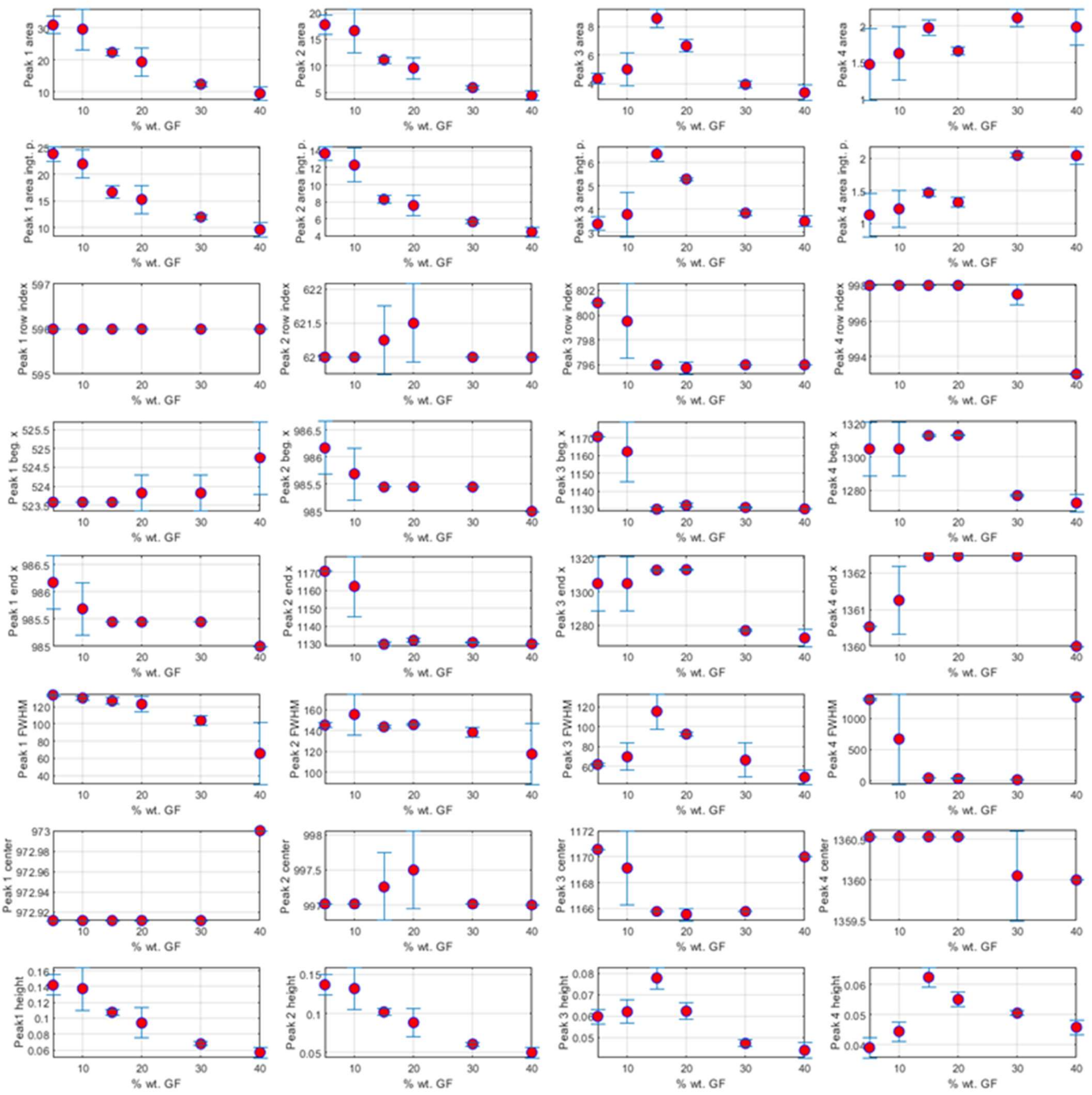

Figure S5. Correlation analysis between selected ATR-FTIR spectral features and mineral filler content (Glass Fiber, GF), highlighting linear relationships used for ANN input selection.

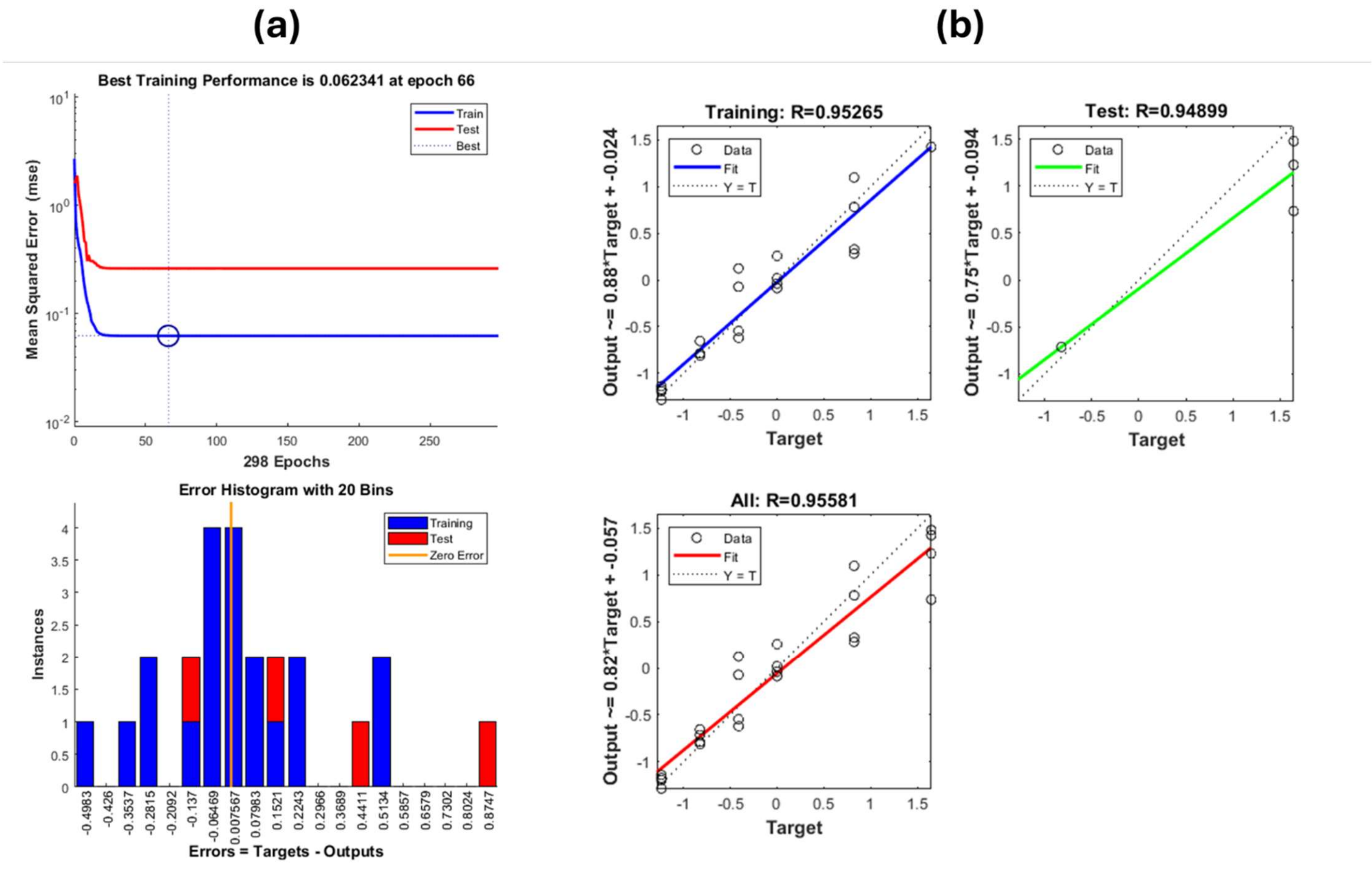

Figure S6. Performance of the ANN for Glass Fiber prediction: training/test MSE, error histogram, and regression plots showing high correlation (R > 0.95) and minimal prediction error.

Table S1. Elemental composition of the polypropylene–talc composite (PP-Talc30%) determined by X-ray fluorescence (XRF) analysis.

Application: Omnian; Normalization factor: 2.822; Minimum He Flow (L/min): 0.69.

Compound	Conc	Unit
Na_2O	606.0	ppm
MgO	18.381	%
Al_2O_3	0.955	%
SiO_2	74.762	%
P_2O_5	1.121	%
SO_4	0.0	ppm
Cl	0.149	%
K_2O	15.9	ppm
CaO	0.566	%
TiO_2	649.0	ppm
V_2O_5	18.7	ppm
Cr_2O_3	103.7	ppm
MnO	241.1	ppm

Fe_2O_3	3.837	%
CoO	189.7	ppm
NiO	86.2	ppm
CuO	4.5	ppm
ZnO	92.1	ppm
Ga_2O_3	9.1	ppm
GeO_2	19.3	ppm
As_2O_3	2.2	ppm
SeO_2	0.0	ppm
SrO	8.1	ppm
ZrO_2	14.7	ppm
CdO	3.1	ppm
In_2O_3	2.9	ppm
SnO_2	104.3	ppm
Sb_2O_3	27.1	ppm
I	0.0	ppm
Cs_2O	31.1	ppm
BaO	49.0	ppm
La_2O_3	0.0	ppm
Eu_2O_3	0.0	ppm
Yb_2O_3	12.9	ppm
IrO_2	0.0	ppm
PbO	9.4	ppm
Rh	0.2	ppm
Re	0.2	ppm

Table S2. Pearson correlation matrices of the chemometric features for each polypropylene composite.

PP-Talc

	CenterP1	CenterP2	HeightP1	HeightP2	WidthP2
CenterP1	1				
CenterP2	0.9	1			
HeightP1	-0.89	-0.98	1		
HeightP2	-0.86	-0.95	0.98	1	
WidthP2	-0.93	-0.96	0.92	0.85	1

PP-$CaCO_3$

	WidthP4	AreaP3	BegP3	AreaP2	HeightP1
WidthP4	1				
AreaP3	0.22	1			
BegP3	0.29	0.9	1		
AreaP2	0.31	0.93	0.86	1	
HeightP1	0.34	0.98	0.9	0.98	1

PP-GF

	HeightP1	BegP2	HeightP3	EndP3	AreaP4
HeightP1	1				
BegP2	0.76	1			
HeightP3	0.62	0.32	1		
EndP3	0.61	0.55	0.77	1	
AreaP4	-0.51	-0.69	-0.14	-0.67	1
